# Creating a 13-year National Longitudinal Cohort of veterans with chronic kidney disease

**DOI:** 10.1186/s12882-019-1430-y

**Published:** 2019-07-03

**Authors:** Mukoso N. Ozieh, Mulugeta Gebregziabher, Ralph C. Ward, David J. Taber, Leonard E. Egede

**Affiliations:** 1 Division of Nephrology, Medical College of Wisconsin, Milwaukee, WI USA; 2 Center for Advancing Population Science (CAPS), Medical College of Wisconsin, Milwaukee, WI USA; 3 Clement J. Zablocki Veterans Affairs Medical Center, Milwaukee, WI USA; 4 Ralph H. Johnson Department of Veterans Affairs Medical Center, Health Equity and Rural Outreach Innovation Center, Charleston, SC USA; 5 Department of Public Health Sciences, Medical University of South Carolina, Charleston, SC USA; 6 Department of Surgery, Medical University of South Carolina (MUSC), Charleston, SC USA; 7 Division of General Internal Medicine, Medical College of Wisconsin, 8701 Watertown Plank Road, Milwaukee, WI 53226-3596 USA

**Keywords:** Veterans, CKD, Kidney disease

## Abstract

**Background:**

The development of large-scale chronic kidney disease (CKD) cohorts within the Veterans Affairs (VA) system has been limited by several factors, including the high proportion of missing race data etc. The goal of this study is to address the limitations of prior studies by creating a large cohort utilizing robust KDIGO recommendations for identifying and staging CKD.

**Methods:**

Multiple patient and administrative files from the Veterans Health Administration (VHA) National Patient Care were linked to create a national cohort of Veterans with chronic kidney disease (CKD) between January 2000 – December 2012; patients identified during this period were followed until 2015. CKD was defined for stages 1 through 5 if markers of kidney damage, specifically proteinuria, were present for at least 3 months. Estimated glomerular filtration rate (eGFR) values were calculated based on serum creatinine levels and the patient’s age, gender, and race using both the Modification of Diet in Renal Disease (MDRD) and Chronic Kidney Disease Epidemiology Collaboration (CKD-EPI) formulas.

**Results:**

About 50 million observations were collected that supported a CKD diagnosis during the study period; these observations corresponded to 3,051,001 unique veterans; 80.9% were non-Hispanic white (NHW), 13.4% were non-Hispanic black (NHB), 3.6% were Hispanic, and 2.0% were in other groups. The mean age 76.7, about 97% were male and 50.2% died prior to January 2016. Among those with stage 3, 12.3% progressed to stage 4, 21.6% of those with stage 4 progressed to stage 5. We found that eGFR values calculated from serum creatinine levels identified about 98% of all patients, while about 11.4% of patients could be identified through ICD-9 codes; only 6.4% could be identified through both sources.

**Conclusion:**

This 13-year national cohort provides an important resource for answering numerous research questions in the future such as racial/ethnic disparities questions, tracking health service utilization, medication adherence, cost and health outcomes in veterans with CKD.

## Background

Chronic kidney disease (CKD) is defined as structural or functional abnormalities of the kidney, with either presence of markers of kidney damage and or decreased glomerular filtration rate (GFR) for > 3 months [1]. CKD is a public health burden [2, 3] and imposes a huge economic burden on individuals affected, their families and the country at large [4–6]. Thirty million US adults are estimated to have CKD and it was ranked the 9th leading cause of death in the US in 2015 [7].

Veterans have approximately 34% higher CKD prevalence than the general population [8], which has been attributed to the significant multi-morbidity and higher mean age in this group. The Veterans Administration health system – the largest U.S. integrated health care system - provides a unique setting to study and monitor progress towards improving the health of people with CKD.

Previous studies have used several CKD definitions to form cohorts [9–12], and these all differed from the current guidelines for disease classification contained in the Kidney Disease: Improving Global Outcomes (KDIGO) 2012 Clinical Practice Guideline for the Evaluation and Management of Chronic Kidney Disease [1]. In some cases, studies relied on the Modification of Diet in Renal Disease (MDRD) equation [13] rather than the Chronic Kidney Disease Epidemiology Collaboration (CKD-EPI) equation [14]. One evaluated normal kidney function using estimated glomerular filtration rate (eGFR) values alone without considering albuminuria; another included a definition for CKD based on International Classification of Diseases (ICD) diagnostic codes.

The development of large-scale CKD cohorts within the Veterans Affairs (VA) system has been limited by several factors, including the high proportion of missing race data, short cohort entry windows, exclusion of Hispanics or women and failure to include markers of kidney damage for early stage kidney disease as recommended by the KDIGO 2012 guidelines [1]. The goal of this study is to address the limitations of prior studies by creating a large cohort utilizing robust KDIGO recommendations for identifying and staging CKD.

## Methods

### Source of Data

**Fig. 1:**
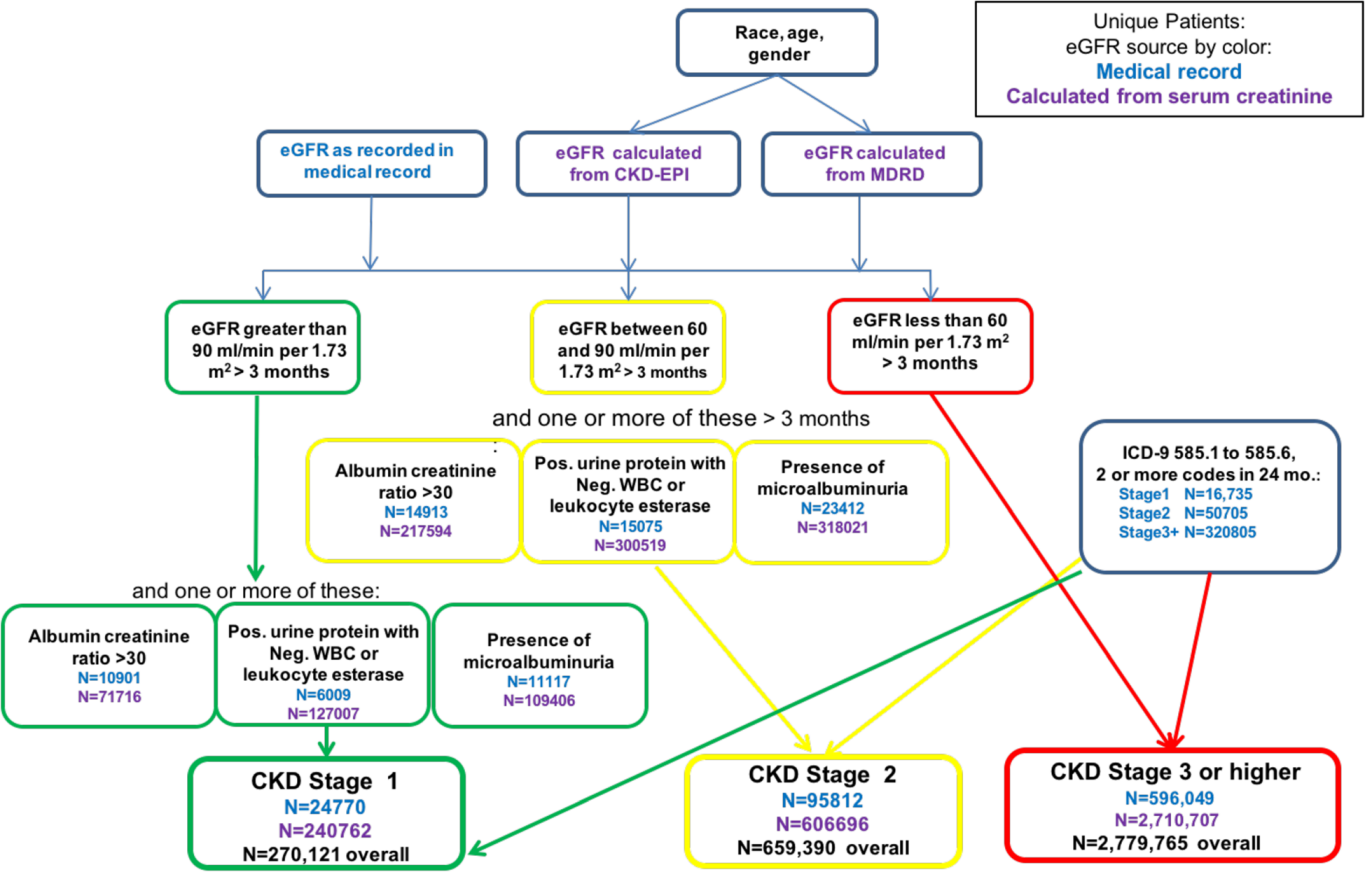
CKD cohort formation based on (1) eGFR values recorded in the patient’s medical record (numbers of unique patients in blue), (2) eGFR values calculated from serum creatinine levels (numbers of unique patients in purple) using the MDRD and CKD-EPI equations, and (3) based on ICD-9-CM codes. Note that a given patient was likely identified by several methods and may appear in multiple CKD stages

Multiple patient and administrative files from the Veterans Health Administration (VHA) National Patient Care were linked [15] to create a national cohort of Veterans with chronic kidney disease (CKD) from January 2000 – December 2012; patients identified during this period were followed until 2015. Figure 1 provides an overview of cohort formation, which closely follows the KDIGO 2012 definition [1]. CKD was defined for stages 1 through 5 when the following conditions were present for at least 3 months: Stage 1: an estimated glomerular filtration rate (eGFR) > = 90 ml/min per 1.73 m^2^ with urine albumin creatinine ratio > 30 mg/g or presence of positive urine protein on dipstick (with negative WBCs or leukocyte esterase) or presence of microalbuminuria; Stage 2: eGFR > = 60 and < 90 ml/min per 1.73 m^2^ with urine albumin creatinine ratio > 30 mg/g or presence of positive urine protein on dipstick (with negative WBCs or leukocyte esterase) or presence of microalbuminuria; Stage 3: eGFR > = 45 and < 60 ml/min per 1.73 m^2^; Stage 4: eGFR > = 15 and < 30 ml/min per 1.73 m^2^; Stage 5: eGFR< 15 ml/min per 1.73 m^2^. In addition, patients who had two or more International Classification of Diseases, ninth revision (ICD-9-CM) codes for CKD stages 1 through 5 (codes 585.1 through 585.6) within a 24-month window were included, including the 24 months prior to initiation of the cohort. Patients under age 18 or with heart, liver or lung transplants were excluded. Estimated GFR values were calculated based on serum creatinine levels and the patient’s age, gender, and race using both the Modification of Diet in Renal Disease (MDRD) equation [13] - GFR (mL/min/1.73 m^2^) = 175 × (Scr)-1.154 × (Age)-0.203 × (0.742 if female) × (1.212 if African American) and Chronic Kidney Disease Epidemiology Collaboration (CKD-EPI) equation [14] - GFR = 141 × min (Scr/κ, 1)α × max (Scr/κ, 1)-1.209 × 0.993Age × 1.018 [if female] × 1.159 [if black] equations to support future comparisons of the two methods.

Since CKD disproportionately affects certain racial and ethnic minority groups [16], we took steps to minimize the fraction of missing race/ethnicity values. We developed an optimized algorithm based on the full information maximum likelihood approach [17], and used multiple VHA Corporate Date Warehouse (CDW) and Medicare sources to reduce the fraction of missing race data to less than 1%.

We collected each patient’s ICD-9 codes over the entire study period, and determined the 31 Elixhauser comorbidities as enhanced by Quan [18].

Our Institutional Review Board (IRB) and local VA Research and Development committee approved the study. Waiver of patient consent was obtained from our institutional IRB, since the study was based on existing data.

### Study population

Cohort entry was from January 2000 – December 2012; patients identified during this period were followed until loss to follow-up, death, or December 2015.

### Outcome measure

The primary outcome was the proportion of patients that died and the proportion that progressed across the CKD stages within the study’s time frame. Patients who were alive on 31 December 2015 were censored.

#### Demographic variables

This included: 1) Age treated as continuous; 2) Gender treated as nominal; 3) Marital status classified as divorced, single, widowed, or married. 4) Race or ethnicity categorized as Non Hispanic White (NHW), Non Hispanic Black (NHB), Hispanic, and ‘Other’ categories. 5) Veterans Administration geographic regions [1 through 5]. 6) Location of residence (Urban, Rural, Highly Rural) was based on Rural Urban Commuting Area (RUCA) codes which were derived from patient-level, residential zip code information. 7) Percent of service-connected disability, representing the degree of disability due to illness or injury that was aggravated by or incurred in military service, was dichotomized (< 50% =0; ≥50% = 1).

#### Medical comorbidity measure

Medical comorbidities were defined based on the Quan enhanced ICD-9-CM version of the Elixhauser Comorbidity Index [18]. Each was determined based on each patient’s unique ICD-9 codes recorded during the study’s timeframe. The total number of comorbidities was then categorized as 3 or less, 4–5, 6–7, and 8 or more. The Elixhauser comorbidity for renal failure was excluded to avoid collinearity.

### Statistical analysis

Descriptive statistics (means for continuous and proportions for categorical variables) were computed. We estimated progression through the various CKD stages using proportions and calculated median follow up in each stage as well as proportion that died across each stage using descriptive statistics. All analyses were performed in SAS 9.4 (SAS Institute, Inc., Cary NC).

## Results

About 50 million observations were collected that supported a CKD diagnosis during the study period; these observations corresponded to 3,051,001 unique patients. Table 1 provides a summary of the demographic characteristics and important comorbidities for the population, of which 80.9% were non-Hispanic white (NHW), 13.4% were non-Hispanic black (NHB), 3.6% were Hispanic, and 2.0% were in other groups. About 97% was male; the mean age 76.7, and 50.2% died prior to January 2016. Table 2 provides a distribution of patients by their median CKD stage in the last observed year; mortality rates varied between 29 to 81% for stages 1 through 5, respectively. The median follow-time was 104 months overall, or 8.7 years. Table 3 summarizes progression from a given CKD stage to a higher stage with 4.2, 3.5 and 3.1% of patients with CKD stage 1, 2 and 3 respectively at baseline progressing CKD stage 5. While 12.3% of those with stage 3 progressed to stage 4, 21.6% of those with stage 4 progressed to stage 5.

**Table 1:** Baseline Characteristics of Cohort

Sample size (*n*) = 3,051,001	
Mean age (std. dev.)	76.7 (11.0)
Married (%)	57.8
Gender (% female)	3.7
Race (%)	
Non-Hispanic black	13.4
Hispanic	3.6
Other	2.0
Non-Hispanic white	80.9
VA Region (%)	
1 Atlantic	23.2
2 Southeast	20.5
3 Upper Midwest	25.8
4 Central West	15.6
5 Southwest	14.9
Rural-Urban (%)	
Urban	71.0
Rural	28.0
Insular Islands	1.0
*Service-related disability (> 50%)	22.7
Mortality (%) (prior to 1/1/2016)	50.2
Comorbidities	
Hypertension, uncomplicated (%)	90.3
Diabetes with complications (%)	27.1
Diabetes, uncomplicated (%)	48.7
Peripheral vascular disorders (%)	29.0
Chronic pulmonary disease (%)	42.6
Congestive heart failure (%)	27.7
Hypertension, complications (%)	21.2
Kidney transplant (%)	0.5
Medical Comorbidities (%)	
3 or less	24.0
4–5	25.8
6–7	20.9
8 or more	29.4

*Percent of service-connected disability represents the degree of disability due to illness or injury that was aggravated by or incurred in military service

**Table 2:** Distribution of patients by median CKD stage in last observed year

Stage	1	2	3	4	5	Total
Alive	97,923	203,317	1,172,346	31,692	13,699	1,518,977
Dead	40,388	105,338	1,188,259	136,324	61,715	1,532,024
Total	138,311	308,655	2,360,605	168,016	75,414	3,051,001
Mortality (%)	29.2	34.1	50.3	81.1	81.3	50.2
Median follow time (months)	85	98	109	84	80	104

**Table 3:** CKD progression during the study period (2000–2012)

For those in Stage	Percentage who eventually reached CKD stage:			
	2	3	4	5
1	74.9	32.1	8.0	4.2
2		59.1	10.3	3.5
3			12.3	3.1
4				21.6

We found that eGFR values calculated from serum creatinine levels identified about 98% of all patients with CKD, while about 11.4% of patients could be identified through ICD-9 codes; only 6.4% could be identified through both sources. For patients with CKD stages 1 and 2, less than 1% could be identified through both sources.

## Discussion

This 13-year CKD cohort of Veterans overcomes the limitations of previous cohorts by utilizing the most recent KDIGO guidelines and by including patients at all stages of disease regardless of race or gender. Because it captures a large group over a long period, patient trajectories from early to late stage disease can be analyzed. This work will enable numerous follow-on studies concerning health disparities and treatment effects especially in older people with CKD.

The importance of utilizing the most recent KDIGO guidelines in CKD staging cannot be overemphasized. Studies show that the degree of proteinuria and CKD stage impact cardiovascular and overall health outcomes [19–21]. Yet, there is no CKD cohort for Veterans that utilizes the KDIGO recommendations for CKD staging. Our study cohort demonstrates the importance and impact of CKD staging on health outcomes. For example, the overall mortality rate in our CKD cohort was 50%, but at stages 4 and 5, significantly higher mortality rates were observed (81%). Studies have also shown that older people with CKD are at a higher risk of CKD complications as opposed to CKD progression [22]. This could explain why a low percentage of patients in our study with CKD stage 1, 2 or 3 at baseline progressed to CKD stage 5 during the last observed year.

By including several factors not included in the KDIGO 2012 definitions, we will be able to examine their performance in future studies. For example, the KDIGO definitions generally recommend use of the CKD-EPI equation to calculation eGFR; we also included calculations based on the MDRD equation. Though not mentioned in the KDIGO guidelines, we also used ICD-9 codes to identify patients since this was a commonly-used method in numerous previous studies. We showed here that ICD-9 codes identified far fewer patients compared with calculated eGFR values. This may indicate that patients with CKD could have other conditions that were the primary reason for visits, and thus CKD-related codes were less likely to appear in their records. In addition, this highlights some of the pitfalls of relying on ICD-9 codes especially within the VA system where there is a higher likelihood of imprecise ICD-9 coding since they do not rely on coding to generate revenue. More importantly, it underscores the essence of using eGFR to identify patients with CKD in kidney disease research.

The significant proportion of Veterans with CKD imposes a tremendous economic and quality of life burden. It emphasizes the need for research focused on understanding the cause of low eGFR in older people especially those with high albuminuria. In addition, our cohort will be an important resource for answering future research questions and also for identifying barriers to diagnosis and treatment. It will also allow effective tracking of health service utilization, cost and health outcomes in this disease population. It will ultimately set the stage for the development and improvement of health care policies geared towards improving health and health outcomes, eliminating health and racial disparities in Veterans with CKD.

Our cohort has several limitations. First, it has limited generalizability since only 4% of the cohort was female (122,040 individuals) and the higher mean age of the cohort. However, the population is large enough to provide reasonable estimates. Furthermore, substantial information on the health of people with CKD can be gleaned from the VHA system data since it is the largest integrated health system in the US with ability to monitor unique patients longitudinally. Second, selection bias i.e. decrease in prevalence estimates, associated with identifying individuals with CKD stage 1 and 2 exists since urine protein excretion is more likely to be checked in high risk individuals. However, creation of this cohort would allow for identification of guideline adherence especially among high risk individuals and provides greater insights to health care providers and policymakers. For example, KDIGO guidelines recommend the use of angiotensin converting enzyme inhibitors or angiotensin II receptor blockers for diabetic CKD patients with proteinuria. Adherence to such a recommendation can be tracked overall or by race using this cohort if merged with VA pharmacy benefits management database. Third, there is a higher likelihood of imprecise ICD-9 coding within the VA system since they do not rely on coding to generate revenue. However, the majority of patients with CKD were identified using eGFR estimation. Fourth, eGFR estimation was based on CKD-EPI and MDRD and the most severe (lowest) eGFR result was used for CKD classification. While this approach optimizes identification of subjects with CKD, it may misclassify a small proportion of individuals. However, sensitivity analysis suggest this is the optimal approach for case identification in large electronic records datasets such as ours.

## Conclusion

In summary, this is the first large-scale CKD Veteran cohort based on the most recent KDIGO guidelines. It captures 13 years of patient history and provides an important resource for answering numerous research questions in the future such as racial/ethnic disparities questions, tracking health service utilization, medication adherence, cost and health outcomes in veterans with CKD.

## Data Availability

The data that support the findings of this study are available from VAMC but restrictions apply to the availability of these data, which were used under license for the current study, and so are not publicly available. Data are however available from the authors upon reasonable request and with permission of the VAMC.

## Abbreviations

CKD: Chronic Kidney Disease
CKD-EPI: Chronic Kidney Disease Epidemiology Collaboration
eGFR: Estimated Glomerular Filtration Rate
ICD-9-CM: International Classification of Diseases, Ninth Revision, Clinical Modification
IRB: Institutional Review Board
KDIGO: Kidney Disease: Improving Global Outcomes
MDRD: Modification of Diet in Renal Disease
NHB: Non Hispanic Black
NHW: Non Hispanic White
RUCA: Rural Urban Commuting Area
VA: Veterans Affairs
VAMC: Veterans Affairs Medical Center
VHA: Veterans Health Administration
